# Long-Term Effects of Neonatal Exposure to Hydroxylated Polychlorinated Biphenyls in the BALB/cCrgl Mouse

**DOI:** 10.1289/ehp.7735

**Published:** 2005-04-20

**Authors:** Jeanelle M. Martinez, L. Clifton Stephens, Lovell A. Jones

**Affiliations:** 1 Department of Gynecologic Oncology and; 2 Department of Veterinary Medicine and Surgery, University of Texas, M.D. Anderson Cancer Center, Houston, Texas, USA

**Keywords:** BALB/cCrgl mouse, estrogenicity, female reproduction, hydroxylated polychlorinated biphenyls, OH-PCBs, tumorigenicity

## Abstract

The neonatal mouse model has been a valuable tool in determining the long-term effects of early exposure to estrogenic agents in mammals. Using this model, we compared the effects of 2′,4′,6′-trichloro-4-biphenylol (OH-PCB-30) and 2′,3′,4′,5′-tetrachloro-4-biphenylol (OH-PCB-61) as prototype estrogenic hydroxylated PCBs (OH-PCBs) because they are reported to exhibit relatively high estrogenic activity both *in vivo* and *in vitro*. The purpose of this study was to examine the relationship between estrogenicity and carcinogenicity of OH-PCB congeners. The OH-PCBs were tested individually and in combination to determine whether effects of combined OH-PCBs differed from those of these OH-PCBs alone. We evaluated the long-term effects of neonatal exposure to OH-PCBs with treatment doses that were based on the reported binding affinity of specific OH-PCB congeners to estrogen receptor α. BALB/cCrgl female mice were treated within 16 hr after birth by subcutaneous injections every 24 hr, for 5 days. The mice treated with OH-PCB-30 (200 μg/day) or 17β-estradiol (5 μg/day) showed similar increased incidences of cervicovaginal (CV) tract carcinomas (43% and 47%, respectively). In addition, when mice were treated with OH-PCBs as a mixture, a change in the type of CV tract tumor was observed, shifting from predominantly squamous cell carcinomas to adenosquamous cell carcinoma. From our results, we conclude that the individual OH-PCBs tested were estrogenic and tumorigenic in mice when exposed during development of the reproductive tract. These data support the hypothesis that mixtures may act differently and unexpectedly than do individual compounds.

Evidence that estrogen acts as a gynecologic carcinogen comes from cases of adenocarcinoma and nonneoplastic abnormalities of the genital tract in females who had been exposed to diethylstilbestrol (DES) *in utero* (Herbst et al. 1971; Robboy et al. 1977). The subsequent cases of cancer and other gynecologic abnormalities in females exposed to DES *in utero* helped to establish the paradigm that a developing fetus is sensitive to compounds tolerated by adults. This paradigm led researchers to reexamine the potential effects of endocrine-disrupting chemicals in human and wildlife species (Gray 1998; Santodonato 1997; Semenza et al. 1997; Zou and Fingerman 1997).

In mice, neonatal exposure to potent natural and synthetic estrogens results in the development of cervicovaginal (CV) tumors, some of which resemble tumors in human females exposed to DES *in utero* (Bern et al. 1975; Bern and Talamantes 1981). Most significantly, these tumors in the mouse model, like those in women transplacentally exposed to DES, are dependent on the dose and time of exposure to the estrogen. Correlation of estrogenicity of DES with carcinogenicity has been demonstrated in the mouse uterus but requires an endogenous source of estrogen for both tumor initiation and progression (Newbold et al. 1990). 17α-Estradiol is a natural estrogen that binds weakly to the estrogen receptor (ER). In mice, exposure to 17α-estradiol during a critical period of reproductive tract development leads to subsequent gynecologic malignancies (Hajek et al. 1997). These studies exemplify that various abnormalities in long-term studies are dependent on when mammals are exposed to a natural or synthetic estrogen.

Although there are many known estrogenic chemicals, we were interested specifically in estrogenic hydroxylated polychlorinated biphenyls (OH-PCBs) because the role they play in breast cancer is controversial and uncertain (Adami et al. 1995; Aschengrau et al. 1998; Krieger et al. 1994). PCBs belong to a class of organochlorine synthetic chemicals that have up to 209 congeners or configurations possible, depending on the number and location of chlorines on the molecule. These PCBs vary in the number of chlorine atoms present, which ranges from 1 to 10, as well as their position on the two benzene rings. The relative toxicity of PCBs depends upon chemical characteristics such as chlorination, hydrophobicity, and planarity (Brouwer et al. 1999). The biologic activity of PCBs is generally classified as dioxin-like or nondioxin-like depending on their mechanism of action. Dioxin-like compounds assume a coplanar configuration with chlorine atoms on the *meta* or *para* benzene position and have a high binding affinity to the aryl hydrocarbon receptor (AhR). Through activation of the AhR, they elicit dioxin-like biochemical and toxic responses. Nondioxin-like chemicals assume a noncoplanar configuration with chlorine atoms on the *ortho* benzene position and bind with variable affinities to steroid hormone receptors. Certain PCBs found in the environment have been shown to be are estrogenic; for example, Hansen et al. (1995) demonstrated that landfill-associated extracts containing PCBs are uterotropic in prepubertal rats. PCB congeners that are capable of binding to the ER can induce the following estrogen-related effects in rodents: increased uterine wet weight, increased glycogen content, prolonged estrous cycle, and proto-oncogene expression (Ecobichion and MacKenzie 1974; Gellert 1978; Korach et al. 1988). 4-OH-PCBs are the major metabolites of PCBs. They are found in human and wildlife blood and appear to persist and bioaccumulate (Bergman et al. 1994; Hovander et al. 2002; Li et al. 2003). 4-OH-PCBs are formed by an arene oxide intermediate catalyzed by phase I cytochrome P450 enzymes. However, the toxicologic impact of the OH-PCBs and their adverse effect in humans are not well characterized. The placental transfer of OH-PCBs has been recently established (Soechitram et al. 2004), suggesting that these PCB metabolites could have adverse effects during developmental exposure. OH-PCBs have been shown to be antiestrogenic and estrogenic and to bind to the ER and to the thyroid hormone receptor, and they are, in general, endocrine-disrupting chemicals (Arulmozhiraja et al. 2005; Connor et al. 1997; Kitamura et al. 2005; Korach et al. 1988).

The goal of this study was to determine if neonatal exposure to the estrogenic chemicals 2′,4′,6′-trichloro-4-biphenylol (OH-PCB-30) and 2′,3′,4′,5′-tetrachloro-4-biphenylol (OH-PCB-61) results in carcinogenicity. The positions of the chlorines for these two PCBs are indicated in Figure 1. The OH-PCBs are the 4-hydroxylated metabolites of parent PCB-30 and PCB-61. We chose these PCB congeners because they have known estrogenic activity and their binding affinity to the ER is reported in the literature (Table 1). Investigations of early-life-stage exposure to polychlorinated biphenyls (PCBs) are warranted because these organochlorine chemicals and their metabolites readily cross the placenta to the fetus in both humans and rodents and are transferred through breast milk to the newborn (Ando 1978; Ando et al. 1985, 1986). There is a growing database on developmental effects for endocrine-disrupting chemicals with multiple end points, including cancer. In this study, we examined the neonatal effects of OH-PCBs. Although the specific OH-PCBs investigated in this study may not occur in the environment, they are sound prototypes for estrogenic OH-PCBs that bind to ER-αand elicit estrogen-mediated responses.

## Materials and Methods

### Chemicals.

All chemicals were of the highest grade available. 17β-Estradiol (E_2_) was purchased from Sigma Chemical Co. (St. Louis, MO). Both OH-PCB-30 and OH-PCB-61 were generously provided by S. Safe (Texas A&M University, College Station, TX). These OH-PCBs were synthesized and purity confirmed as described previously (Safe et al. 1995). For this study, E_2_ and the OH-PCBs were dissolved in 1 mL 100% ethanol and warmed to dissolve the chemical. Sesame oil was added to obtain the desired concentrations for 20-μL subcutaneous injections. Ethanol was then evaporated using nitrogen gas while keeping the solution warm to prevent recrystallization. OH-PCB doses used in this study are based on their reported respective binding affinity to ER-α. E_2_ (5 μg/day) was used as a predictive dose because the frequency of CV tumors in BALB/cCrgl mice neonatally exposed to E_2_ is approximately 50% (Jones and Bern 1979). To test for interactive effects, doses were selected using the high dose of OH-PCB-30 as a basis of comparison because it has a higher binding affinity to ER-α.

### Animals.

Mice were handled according to the Guide for the Care and Use of Laboratory Animals (Institute of Laboratory Animal Resources 1985), and the Institutional Animal Care and Use Committee approved all procedures performed on animals. Adult mice were fed Purina Rodent Chow 5001 (Alies Seed, Houston, TX). Pregnant female BALB/cCrgl mice were purchased from Harlan Sprague Dawley (Indianapolis, IN). The inbred BALB/cCrgl strain was used because it has a low mammary tumor incidence and its response to E_2_ treatment during neonatal development is well documented. Beginning within 16 hr after birth, female pups were pooled from several litters and distributed four or five pups per mother per cage. Each cage was then given five daily subcutaneous injections with 20 μL sesame oil alone, 5 μg E_2_, 2.5 μg E_2_ plus 100 μg OH-PCB-30, 20 μg OH-PCB-30, 200 μg OH-PCB-30, 40 μg OH-PCB-61, 400 μg OH-PCB-61, 10 μg OH-PCB-30 plus 10 μg OH-PCB-61, or 100 μg OH-PCB-30 plus 100 μg OH-PCB-61 (Table 2). Animals were weaned 21 days of age. Mice were examined daily for premature vaginal opening for the first 35 days of life and checked monthly with blunt forceps to detect concretions (calcium carbonate deposits in the vagina that are a result of malformation of the urogenital tract in developmentally estrogenized animals). When concretions were found, they were removed. All mice that survived to 20 months of age were sacrificed by CO_2_ fixation. Tissues were dissected and fixed in 10% buffered formalin for at least 24 hr before being embedded in paraffin. Paraffin-embedded blocks were serially sectioned and stained with hematoxylin and eosin (H&E).

### Statistical analyses.

We used one-way analysis of variance to assess differences in body weight, uterine weight, and vaginal opening. Pairwise comparisons of each experimental group versus sesame oil control were made by Tukey-HSD (honest significant difference) tests. Survival comparisons were made by Wilcoxon rank sum tests. The proportions of animals with malignant tumors were compared by Fisher exact tests. Animals that died before the appearance of the first tumor were excluded from the analysis.

## Results

### Gross observations.

A biologic index of sexual maturity can be visually assessed by day of vaginal opening (DVO). The DVO was significantly shorter in mice given E_2_ alone, E_2_ plus OH-PCB-30 (200 μg), OH-PCB-61 (40 and 400 μg), and the mixture OH-PCB-30/61 (100/100 μg; Table 2). There was a dose-dependent effect with the higher dose yielding the shortest DVO. The lower doses of OH-PCBs had a DVO similar to that in control mice. Body weight was significantly decreased in mice given 5 μg E_2_. Mortality was increased in mice given OH-PCB at high doses (*p* < 0.05; Table 2).

### Tumor incidence.

Tumor incidences are summarized in Table 3. The only tumor seen in control mice was one malignant lymphoma. The incidence of malignant tumors was significantly greater in all groups exposed to E_2_ and/or PCB than in controls. Among mice given E_2_ alone, incidence of CV tract carcinomas was 43% (16 of 37), and for any tumor, 49% (18 of 37; some mice had more than one type of tumor detected). We detected other tumors that are commonly observed in mice neonatally treated with E_2_, including cholangiocarcinoma of the gallbladder and granulosa cell tumor. The E_2_-treated mice also had one incidence of bronchoalveolar adenoma of the lung. Among mice given E_2_ plus OH-PCB-30, incidences of CV tract carcinomas (47%; 9 of 19) and granulosa cell tumors (15%; 3 of 19) were significantly increased. In addition, there was one reticulum cell sarcoma detected in mice treated with E_2_ plus OH-PCB-30.

In mice given the high dose of OH-PCB-30 (200 μg/pup/day), the incidences of CV tract carcinomas and granulosa cell tumors were 45% (10 of 22) and 14% (3 of 22), respectively; one mouse was found with cholangio-carcinoma. Incidences of tumors in the low-dose OH-PCB-30 mice (20 μg/pup/day) were as follows: CV tract carcinomas, 6% (2 of 33); mammary gland adenocarcinoma, 15% (5 of 33); and bronchoalveolar adenoma/ carcinoma, 9% (3 of 33).

In mice neonatally treated with 400 μg OH-PCB-61, we found a 20% incidence of CV tract carcinomas (5 of 24), 4% incidence of mammary gland adenocarcinoma (1 of 24); and an 8% incidence of hemangiosarcoma (2 of 24). From all of the treatment groups, we observed one animal with hepato-cellular carcinoma—a mouse treated with 400 μg OH-PCB-61. In mice treated with the low dose of OH-PCB-61 (40 μg), tumor incidences were as follows: CV tract carcinomas, 13% (4 of 30); granulosa cell tumor, 10% (3 of 30); mammary gland tumors, 13% (4 of 30); and hemangiosarcomas, 7% (1 of 30). In mice given 200 μg of the mixture (OH-PCB-30/61), the incidences of neoplasms detected were as follows: CV tract carcinomas, 38% (8 of 21); granulosa cell tumor, 10% (2 of 21); malignant lymphomas, 10% (2 of 21); and bronchoalveolar carcinoma, 5% (1 of 21). Incidence rates for mice treated with 20 μg OH-PCB-30/61 were 8% (3 of 36) for CV tract carcinomas, 3% (1 of 36) for granulosa cell tumor, and 8% (3 of 36) for mammary gland carcinomas.

### Interactive effects of chemical mixtures.

The two types of tumors detected in groups administered estrogenic compounds alone and in combination were compared by Fisher exact tests (Table 4). We observed no detectable differences in the overall incidence of CV tract tumors. However, there was a difference in the relative distributions of tumor types. In 8% (3 of 37) of animals treated with E_2_ and in 14% (3 of 22) of animals treated with 200 μg OH-PCB-30, we observed a significant difference between the combined incidences of CV tract adenosquamous cell carcinoma compared with that of animals treated with E_2_/OH-PCB-30 (32%; 6 of 19), as determined using Fisher exact tests. Although it was not statistically significant, there appeared to be a trend for an increased incidence of CV tract development of adenosquamous cell carcinoma versus squamous cell carcinoma when comparing the combined incidence of OH-PCB-30 (14%; 3 of 22) and OH-PCB-61 (8%; 2 of 20) to that of OH-PCB-30/61 (24%; 5 of 21).

## Discussion

In this study, we used the DES neonatal mouse model to evaluate the tumorigenic effects of estrogenic OH-PCBs. The results show that the production of CV tract tumors occurred to a similar degree between 5 μg E_2_ (43%; 16 of 37) and 200 μg OH-PCB-30 (47%; 9 of 19). A rather large number of different tumors were detected in this study, but the tumors with the highest frequency were the CV tract tumors (Table 3). These CV tract tumors were induced by neonatal OH-PCB treatment. A limitation of this study was the number of doses used, but there appeared to be a pattern of increased CV-tract tumors with the higher doses. These data strongly support the theory that relatively weak estrogens can induce tumors in mice when exposure occurs during a critical period of development (Hajek et al. 1997).

The neonatal mouse model has been extensively studied for more than four decades and has proven extremely valuable in assessing human in utero exposure to DES. The defined period for causation of genital tract tumors by natural (17α-estradiol and E_2_) and synthetic (e.g., DES) estrogens occurs during the development of the reproductive tract in both humans and rodents (Hajek et al. 1997). The use of the neonatal mouse model was necessary because, unlike findings in adult-treated rodents (Liehr et al. 1986), an apparent correlation between estrogenicity and carcinogenicity exists in neonatally treated rodents (Newbold et al. 1990, 1997). In addition, species-specific E_2_-mediated tumor induction occurs in different strains of mice. For example, outbred female CD-1 mice are susceptible to uterine tumors, and inbred BALB/cCrgl mice are hormonally susceptible to CV tract tumors (Jones and Bern 1977). E_2_-mediated tumor induction is also age dependent and dose related and, most important, occurs in a tissue-dependent manner (Newbold et al. 1990).

Our experiments were aimed at determining a relationship between estrogenicity and carcinogenicity for estrogenic PCBs. The first indication of the estrogenicity of E_2_ and/or OH-PCBs in the present study was premature vaginal opening (Table 2). OH-PCBs tested alone or in combination facilitated premature vaginal opening in a time frame similar to that of E_2_. Both OH-PCB-30 and OH-PCB-61 have also tested positive for in vivo estrogenicity in juvenile fish and mice (Carlson and Williams 2001; Korach et al. 1988). Like other studies testing interactions, we only found additive effects from the combined chemicals (Carlson and Williams 2001; Ramamoorthy et al. 1997). We found that the highest mortality rates were seen in mice treated with high doses of OH-PCBs, indicating that neonatal exposure to PCBs has a chronic toxic effect because the lethality occurred close to 12 months. Some of the chronic carcinogenic effects attributed to OH-PCB exposure in this study were similar to those known for E_2_, but others, such as tumor formation in organs other than the CV tract, were not. Thus, the tumors seen in E_2_-treated mice reflect the species-specific E_2_-mediated tumor susceptibility of BALB/cCrgl mice. In contrast to findings in the literature that mixtures of PCBs promote hepatocellular carcinoma (Dutch Expert Committee 1995; Mayes et al. 1998; Sleight 1985), a variety of malignant tumors were identified in the OH-PCB–treated mice, but only one mouse developed a hepatocellular carcinoma; thus, the mechanisms are likely to be very different.

The incidence of mammary gland carcinomas was significantly increased to 13% (4 of 30) in mice treated with 40 μg OH-PCB-61. Mammary gland tumors were also detected in mice treated with E_2_ (3%; 1 of 37), 400 μg OH-PCB-61 (13%; 4 of 30), 20 μg OH-PCB-30 (15%; 5 of 33), and 20 μg OH-PCB-30/61 (8%; 3 of 36). Although several published studies support the idea that developmental exposure to PCBs may lead to an increase in breast cancer (Birnbaum and Fenton 2003; Desaulniers et al. 2001; Mayes et al. 1998), the results from the present study are striking in that we detected an increased number of mammary tumors. Historically, BALB/cCrgl mice do not develop mammary gland tumors (Dunn and Green 1963; Mori et al. 1976). We did not find a clear dose-dependent increase in mammary gland tumor responses because there were fewer mammary gland tumors detected in the high-dose OH-PCB-61 mice than in the low-dose OH-PCB-61 mice. Also, we detected no mammary gland tumors in the high dose OH-PCB-30 mice, but 5 were found in the low-dose OH-PCB-30 mice. This effect is probably due to the increased mortality in high-dose groups (Table 2). Unfortunately, no dissections or histologic analysis occurred if animals died on weekends or at night. In addition, the mammary glands were not dissected out from control animals, and the only reason mammary gland tumors were detected at all is because they were visibly obvious.

Effects on mammary growth, lobuloalveolar development, and hyperplastic alveolar nodules as well as dysplasias have been detected (Jones and Bern 1977, 1979) in virgin female BALB/cCrgl mice neonatally treated with estrogen. Mammary tumors have been found in transplantation studies (Medina 1976) where hyperplastic alveolar nodules from 7,12-dimethylbenz[*a*]anthracene-treated mice were placed into the mammary fat pad of virgin BALB/cCrgl mice. The average time for development (6 of 6; 100%) of tumors was 6 months. It has been postulated that the mouse mammary tumor virus (MMTV) is essential for the development of mammary gland tumors. This theory is strongly supported by findings that hormonally neonatally treated mice that have MMTV develop mammary gland tumors (Jones and Bern 1979). It was unfortunate that the mammary gland was not chosen as a target organ, but we did not expect to find mammary gland tumors in treated inbred mice that lack MMTV. The induction of mammary gland tumors by neonatal OH-PCB may be due to the combination of its overall carcinogenicity with its estrogenicity. Future studies using this animal model are necessary to determine the mechanism of action. In humans, the association of PCBs with breast cancer has not been determined. Although exposure to elevated levels of PCBs is still a potential factor in breast cancer (Laden et al. 2002; Wolff and Toniolo 1995), a correlation has not been established (Brown 1987; Higginson 1985; Krieger et al. 1994; Laden et al. 2001).

There are two significant results of this study: the demonstration that OH-PCB congeners are carcinogenic, and that the type of CV tract tumors observed in response to treatment with a mixture was significantly different than from those found after individual OH-PCBs treatment. For both mixture groups (E_2_/OH-PCB-30, and OH-PCB-30/61), we found a lower incidence of CV tract squamous cell carcinomas and elevated incidence of CV tract adenosquamous cell carcinoma. Thus, a shift from squamous to adenosquamous was observed in mice treated with mixtures. This is a very interesting result because it illustrates clearly that the toxic response to mixtures may be different from the toxic response of the individual components of the mixture. Gynecologic epithelial tumors are generally grouped into these two major categories based on whether they are derived from Mullerian epithelium (adenocarcinoma) or squamous epithelium (squamous cell carcinoma) of the urogenital sinus. The adenosquamous carcinoma of the CV tract may be similar to the adenosquamous carcinoma of the lung, which is an example of a heterogeneous tumor (Kanazawa et al. 2000). Adenosquamous carcinomas of the lung and CV tract are similar in clinical outcome: the prognosis is poorer than for patients with either squamous carcinomas or adenocarcinomas (Farley et al. 2003; Hofmann et al. 1994).

The present study supports the hypothesis that neonatal exposure to estrogenic OH-PCBs mimics the ability of E_2_ to induce CV tract tumors in the BALB/cCrgl mouse. For example, there was an increase in CV tumors induced by higher doses of OH-PCB-30 compared with lower doses. In addition, similar molecular and morphologic effects were true to a lesser extent for PCB-61. The dose of OH-PCB-61 was twice that of OH-PCB-30; therefore, a similar incidence of CV tract tumors was expected based on receptor binding affinities. Instead, there was less than half as many: incidence rates for CV tract tumors were 21% (5 of 24) versus 46% (10 of 22) for the high doses of OH-PCB-61 and OH-PCB-30, respectively. This may be a result of toxicity as indicated by higher mortality (Table 2).

Assessing the long-term effects of PCBs is important because the general population is exposed to these chemicals at all stages of human development. In a series of reports, researchers from the Netherlands associated prenatal exposure to PCBs with biologic effects (Huisman et al. 1995; Patandin et al. 1999a, 1999b). Similarly, perinatal exposure to PCBs is linked to a variety of immunologic, neural, and endocrine effects and potentially linked with biologic effects on growth, sexual development, and long-term reproductive health (Weisglas-Kuperus 1998). Perinatal exposure to PCBs has been associated with smaller head circumference and lower birth weight (Fein et al. 1984; Taylor et al. 1989). One study also reported a decrease in penis size in boys born to mothers exposed to PCBs, but this finding may be difficult to interpret because the maternal exposure was to a mixture of PCBs most likely contaminated with similar organochlorines, that is the polychlorinated dibenzo-*p*-dioxins/dibenzofurans (Guo et al. 1995). These studies emphasize the need for testing individual compounds and as compounds in mixtures.

## Conclusion

OH-PCBs induced predominantly mammary gland and CV tract tumors in mice that were exposed during a critical period of development. OH-PCBs induced tumors in other organs, suggesting that the carcinogenic effect is not restricted to estrogen-sensitive organs. These findings suggest that other organs should be examined in future epidemiologic studies with OH-PCBs. Finally, we believe this report is the first to show that a chemical mixture shifts the tumor type from squamous to adenosquamous, suggesting that exposure to a mixture may result in the formation of a more aggressive tumor type.

## Figures and Tables

**Figure 1:**
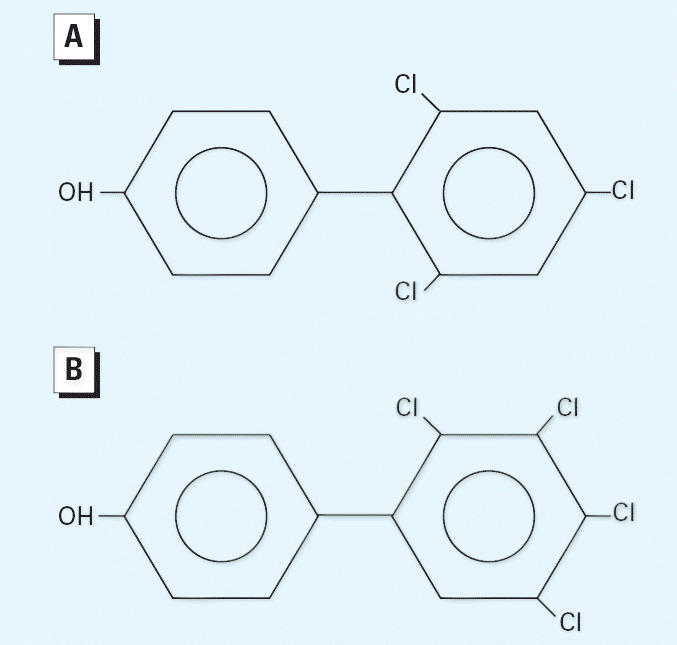
Chemical structures for OH-PCB-30 (*A*) and OH-PCB-61 (*B*).

**Table 1:** Chemical nomenclature, abbreviations, and ER-αbinding.

Chemical name	Abbreviation	C_50_^a^	Observed log IC_50_^b^
17β -Estradiol	E_2_	1	0.837
2′,4′,6′-Trichlorobiphenyl	PCB-30	—	6.77
2′,4′,6′-Trichloro-4-biphenylol	OH-PCB-30	42	2.84
2′,3′,4′,5′-Tetrachlorobiphenyl	PCB-61	—	ND^c^
2′,3′,4′,5′-Tetrachloro-4-biphenylol	OH-PCB-61	95	2.15

a The molar equivalent required to occupy 50% of the mouse uterine ER-αbinding site (Korach et al. 1988).

b The concentration of competitor predicted to cause a 50% reduction in specific binding of radiolabeled 17β-estradiol to calf uterine ER.

c Not detected (ND) at doses tested (Kramer and Giesy 1999).

**Table 2:** Gross observations from neonatally treated BALB/c mice at 20 months of age.

Neonatal treatment (μg/pup/day)	DVO (mean ± SE)	Body weight (g; mean ± SE)	Mortality^a^ (%)	No.^b^
Oil	23.8 ± 0.6	25.0 ± 0.37	9	35
E_2_ (5)	10.5 ± 0.4*	23.0 ± 0.43*	16	43
E_2_ (2.5) plus OH-PCB-30 (100)	10.9 ± 0.4*	24.8 ± 0.50	21	24
OH-PCB-30 (200)	11.1 ± 0.2*	24.6 ± 0.40	31**	32
OH-PCB-30 (20)	24.8 ± 0.4	24.8 ± 0.51	21	39
OH-PCB-61 (400)	12.4 ± 0.4*	24.7 ± 0.40	33**	33
OH-PCB-61 (40)	17.7 ± 0.8*	25.0 ± 0.44	19	31
OH-PCB-30/61 (100 + 100)^c^	12.1 ± 0.4*	25.7 ± 0.62	30**	27
OH-PCB-30/61 (10 + 10)^c^	22.4 ± 0.6	25.3 ± 0.33	18	40

DVO, day of vaginal opening. Pups were treated as described in “Materials and Methods.”

a Percentage of animals that died before the end of the study.

b Number of animals used for study.

c Equal concentrations of OH-PCB-30 and OH-PCB-61 were used as a mixture.

*p < 0.05 versus sesame oil control (Tukey-HSD test).

**p < 0.05 versus sesame oil control (Wilcoxon rank sum test).

**Table 3:** Summary of specific tumor incidence in BALB/c mice treated neonatally and sacrificed at 20 months of age.

	Incidence of tumor type									
Neonatal treatment (μg/pup/day)	ML	H	BA	C	CV	OG	MG	Ot^a^	TNT^b^	No.^c^
Oil	1	0	0	0	0	0	0	0	1	33
E_2_ (5)	0	0	1	2	16**	1	1	0	18**	37
E_2_ (2.5)/OH-PCB-30 (100)	0	0	0	0	9**	3*	0	1	11*	19
OH-PCB-30 (200)	0	0	0	1	10**	3	0	0	12*	22
OH-PCB-30 (20)	0	0	3	0	2	0	5	0	9*	33
OH-PCB-61 (400)	0	2	0	0	5*	0	1	2	11*	24
OH-PCB-61 (40)	2	1	0	0	4*	3	4*	2	15*	30
OH-PCB-30/61 (100 + 100)^d^	2	0	1	0	8*	2	0	2	13*	21
OH-PCB-30/61 (10 + 10)^d^	0	0	0	0	3	1	3	1	8*	36

Abbreviations: BA, bronchoalveolar; C, cholangiocarcinoma of the gallbladder; CV, cervicovaginal tract carcinoma; H, hemangiosarcoma; OG, ovarian granulosa cell tumor; MG, mammary gland carcinoma; ML, malignant lymphoma; OG, ovarian granulosa cell tumor; Ot, other types of tumors not listed; TNT, total number of tumors found in that treatment group. Pups were treated as described in “Materials and Methods.”

a Tumor type occurred in no more than one animal per group.

b Some mice had more than one type of tumor.

c Number of mice diagnosed by H&E staining.

d Equal concentrations of OH-PCB-30 and OH-PCB-61 were used as a mixture.

*p < 0.05 versus sesame oil control (Fisher exact test).

**p < 0.01 versus sesame oil control (Fisher exact test).

**Table 4:** Interactive effects on frequency of carcinoma types in the CV tract.

	Percent frequency		
Neonatal treatment (μg/pup/day)	Total incidence^a^	Squamous	Adenosquamous
E_2_ (5)	16/37^b^	41 (15/37)	8 (3/37)
OH-PCB-30 (200)	10/22^b^	36 (8/22)	14 (3/22)
OH-PCB-61 (400)	5/24	13 (3/24)	8 (2/24)
E_2_ (2.5)/OH-PCB-30 (100)	9/19	16 (3/19)	32 (6/19)*
OH-PCB-30/61 (100 + 100)^c^	8/21	14 (3/21)	24 (5/21)

Pups were treated as described in “Materials and Methods.”

a Total incidence is the number of CV tract tumors per total number of mice treated.

b Some mice had more than one type of CV tract tumor.

c Equal concentrations of OH-PCB-30 and OH-PCB-61 were used as a mixture.

*p < 0.05 versus a combination of E_2_ (5) and OH-PCB-30 (200), Fisher exact test.
